# Correction to “Gout Remission With Pegloticase‐Induced Intensive Urate‐Lowering Therapy: A Post Hoc Clinical Trial Analysis”

**DOI:** 10.1002/acr2.70128

**Published:** 2025-10-06

Klionsky, Y. , Torralba, K. D. , Obermeyer, K. , Padnick‐Silver, L. , Lam, G. , LaMoreaux, B. Gout Remission With Pegloticase‐Induced Intensive Urate‐Lowering Therapy: A Post Hoc Clinical Trial Analysis. ACR open rheumatology, 2025;7(8), e70080. 10.1002/acr2.70080 40810458 PMC12351638

In the affiliation information, the current address of author Lissa Padnick‐Silver was missing from the originally published version of the article. In addition, the author disclosures form was also missing from the article's supporting information. The online version of this article has been corrected accordingly.

We apologize for this error.

